# Non-Traditional Aromatic Topologies and Biomimetic Assembly Motifs as Components of Functional Pi-Conjugated Oligomers

**DOI:** 10.3390/ma3021269

**Published:** 2010-02-23

**Authors:** John D. Tovar, Stephen R. Diegelmann, Patricia A. Peart

**Affiliations:** 1 Department of Chemistry, Johns Hopkins University, 3400 N. Charles St., Baltimore, MD, 21218 USA; E-Mails: diegelmanns@jhu.edu (S.R.D.); ppeart1@jhu.edu (P.A.P.); 2 Department of Materials Science and Engineering, Johns Hopkins University, 3400 N. Charles St., Baltimore, MD, 21218 USA; 3 Institute for NanoBioTechnology, Johns Hopkins University, 3400 N. Charles St., Baltimore, MD, 21218 USA

**Keywords:** conjugated polymers, self-assembly, aromaticity

## Abstract

This article will highlight our recent work using conjugated oligomers as precursors to electroactive polymer films and self-assembling nanomaterials. One area of investigation has focused on nonbenzenoid aromaticity in the context of charge delocalization in conjugated polymers. In these studies, polymerizable pi-conjugated units were coupled onto unusual aromatic cores such as methano[10]annulene. This article will also show how biologically-inspired assembly of molecularly well-defined oligopeptides that flank pi-conjugated oligomers has resulted in the aqueous construction of 1-dimensional nanomaterials that encourage electronic delocalization among the pi-electron systems.

## 1. Introduction

Since the discovery of electrical conductivity in organic molecular solids and conjugated polymers [1,2,3], tremendous interest has evolved with respect to new pi-conjugated organic materials. The development of soluble conjugated polymers has created new possibilities for solution-processed transistors, light-emitting diodes, electrochromics and photovoltaics in some cases operable on large area and flexible substrates [4]. A sound structure-function relationship is important in order to harness the most function out of existing materials as well as to design even higher performing materials. In the context of conjugated polymers, two critical charge transporting regimes have been identified: intrapolymer and interpolymer. Although operable over much different size scales, both regimes help to define the “band structure” and thus the electronic properties of the bulk material. Well-defined conjugated oligomers have served as crucial models for describing both of these regimes. In this report, we will describe recent work showing how one can use unusual aromatics as part of defined oligomers and polymers to increase the effective conjugation length. We will also report a new assembly scheme to achieve strong intermolecular delocalization among pi-conjugated oligomers in aqueous environments.

There typically exists a finite number of repeat units within a conjugated polymer that can be sufficient to describe the bulk electronic properties of the polymer. This is referred to as the effective conjugation length or oligomer extrapolation [5]. Using standard synthetic cross-coupling techniques, several series of conjugated oligomers have been prepared and compared to the corresponding conjugated polymers (e.g. oligo-α-thiophenes as models for polythiophene). It has been shown that a convergence of the optical properties (as determined by UV-vis λ_max_ values) occurs with the long oligomer and the corresponding polymer (typically between 8–12 repeat units) [6]. Therefore, one can conceivably construct a molecularly well-defined oligomer of suitable length that will have known chemical constitution and the same optical properties as the corresponding polydisperse polymer.

The effective conjugation length is modulated by the electronics of the component units as well as by conformational effects imposed among them. The latter involves steric influences that may render a pi-conjugated backbone more or less planar. The former is more subtle and can be illustrated with two prototypical 6 pi-electron fragments: benzene and hexatriene. Neglecting thermodynamic and electronic contributions of the sigma framework, a simple Hückel molecular orbital (HMO) treatment reveals a “bandgap” of 0.89 β for hexatriene and 2 β for benzene (where β is the resonance integral in the HMO calculations). On the other hand, benzene has more resonance stabilization energy when compared to the triene. Although the aromatic provides more stability, it is energetically more costly to disrupt the aromaticity as is required for most conducting polymers in their charged “quinoidal” states. Thus one might conclude that the polymers with the lowest energy gaps between the highest occupied and lowest unoccupied molecular orbitals and with the easiest abilities to ionize would be those consisting of polyenes. Unfortunately, polyacetylene as the prototypical polyene is quite difficult to process for synthetic or device manipulation and is not incredibly stable in the doped state. Therefore, we surmised that unusual aromatics with relatively low resonance stabilization energies might facilitate the delocalization of charge (and increase the effective conjugation length) due to unique electronic structures that could also be viewed as “cyclic polyenes.” 

## 2. Results and Discussion

### 2.1. Methano[10]annulene as a component of pi-conjugated oligomers and polymers

We initiated our explorations with the methano[10]annulene ring system, a remarkable hydrocarbon whose synthesis was developed by Professor Emanuel Vogel in the mid-1960s [7]. Although a variety of more complex electronic molecules have been built from this annulene core over the intervening decades [8,9,10], we were not aware of any studies to incorporate this 10 pi-electron aromatic molecule into conjugated polymers, either for solution or thin-film solid-state measurements. Larger aromatics such as the 14 pi-electron dihydrodimethylpyrene have been included into conjugated polymers but have not been compared in a side-by-side sense to more classical benzenoid aromatics [11,12]. Our motivations were driven by this intellectual curiosity, and for the functional reason that the annulene, having a monocyclic pi-electron delocalization pathway, may not have the electronic desire to form localized aromatic sextets as one could conceive for respective benzenoid fused hydrocarbons (e.g. naphthalene).

**Figure 1:**
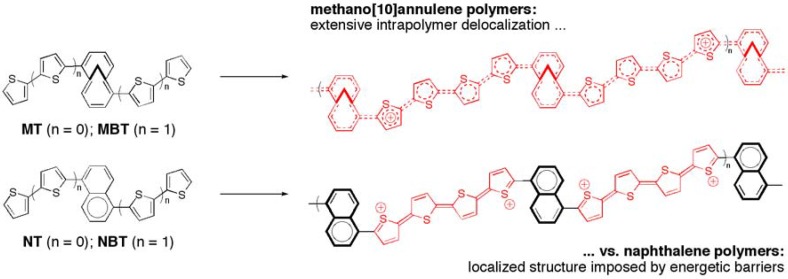
Annulene (**MT and MBT**) and naphthalene (**NT and NBT**) electropolymerizable oligomers (at left), along with the expected polymers formed when n = 1 (at right). The naphthalene is boldened in the aromatic form to emphasize the greater degree of localization observed for naphthyl polymers in their oxidized states.

It is well established that extending the pi-conjugation of a monomer will render the oxidation potential less positive. Therefore, we incorporated thienyl and bithienyl units onto the annulene core to facilitate electrochemical oxidative polymerization (Figure 1) [13,14]. As a comparison 10 pi-electron model, the respective naphthalene monomers were also prepared. The non-linear “Z-shaped” conjugation pathways were chosen in order to require delocalization through the entire pi-system as well as for simple synthetic convenience [15]. The annulene parent hydrocarbon has an absorption onset approximately 30 nm redshifted from naphthalene. In contrast, the respective bithiophene-substituted compounds (**MBT** and **NBT**) had a ca. 100 nm difference! This indicates that the extended conjugation available in the annulene case is magnifying the difference when pi-conjugating substituents are attached.

**Figure 2:**
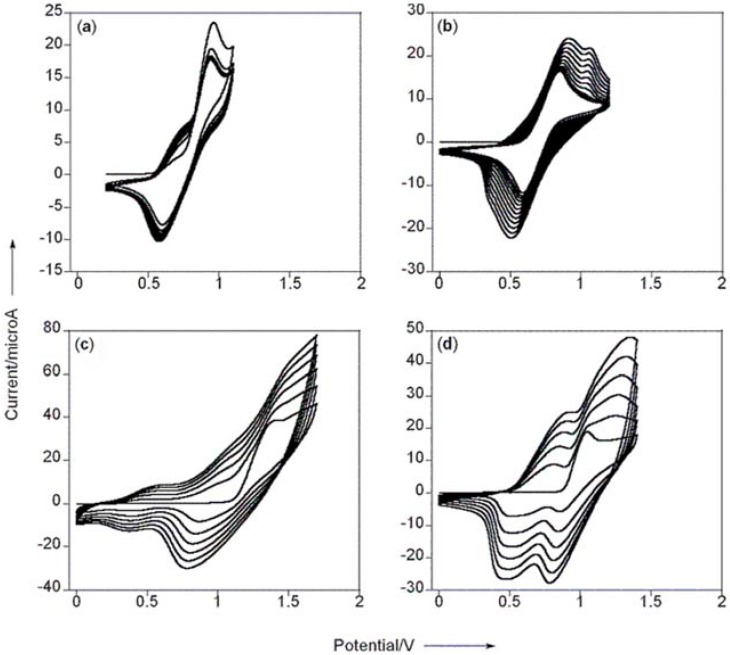
Polymer growth profiles plotted relative to Ag/Ag^+^ for (a) **MT**, (b) **MBT**, (c) **NT** and (d) **NBT** were obtained in 0.1 M *n*-Bu_4_NPF_6_/CH_2_Cl_2_ using a 2 mm^2^ Pt-button working electrode, coiled Pt wire as the counter electrode, and a silver wire reference electrode submersed in 0.01 M AgNO_3_ and 0.1 M TBAP in CH_3_CN. Scan rate = 100 mV/s. Figure taken from reference [14] with permission (Copyright 2008, Wiley-VCH Verlag & Co. KGaA).

Of course, the trends in HOMO-LUMO gaps do not reflect absolute energetics relative to vacuum, but the expectation that molecules with more extended intramolecular delocalization available should undergo oxidation more easily did hold true. The peak oxidation potential (E_pa_) of **MT** was almost 400 mV less positive than **NT**, while the E_pa_ of **MBT** was 200 mV less positive than **NBT** (Figure 2). These differences were magnified after considering the *onset* of oxidation rather than the E_pa_ value. Furthermore, the electrochemical reversibility was much more pronounced for the annulene monomers, also suggesting greater stability of the formed radical cations that retard follow up electrochemical polymerization [16]. This coupled to the fact that the annulene monomers are easier to oxidize than their naphthalene counterparts indicated to us that the enhanced intramolecular delocalization exists both within the neutral and the charged/oxidized annulene monomers.

**Figure 3:**
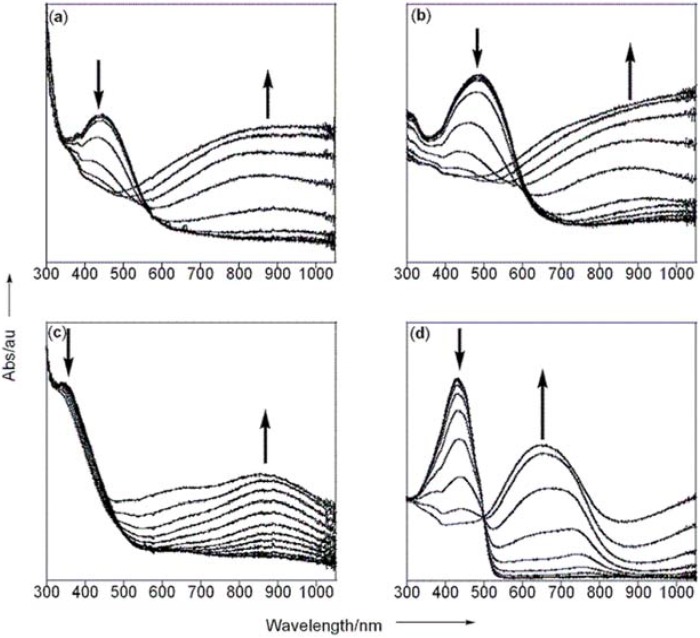
Spectroelectrochemical data obtained on an ITO electrode for (a) poly(**MT**) between 0–1.1 V, (b) poly(**MBT**) between 0–1.1 V, (c) poly(**NT**) between 0–1.2 V and (d) poly(**NBT**) between 0–1.3 V. Other conditions as listed in Figure 2. Figure taken from reference [14] with permission (Copyright 2008, Wiley-VCH Verlag & Co. KGaA).

Spectroelectrochemical data provided the most convincing illustration of this effect being manifested in polymeric materials. Polymerization on transparent indium tin oxide (ITO) electrodes enabled the observation of electronic changes that occur as the polymer is progressively oxidized (or “doped”) into conductive states [17]. These conductive states typically correspond to the quinoidal electronic structure that is delocalized over several repeat units of the polymer chain. As the annulene polymers were oxidized, the absorptions for the neutral polymers decreased in intensity and were accompanied by lower energy absorptions that correspond to quinoidal species (Figure 3a,b). These low energy absorptions were quite broad and featureless. Quite different responses were noted for the naphthalene containing polymers (Figure 3c,d). For poly(**NT**), the neutral absorption persisted during the evolution of more structured low-energy activity. Poly(**NBT**) showed the more classic decrease in neutral absorption with increased applied potential, but now with a very pronounced absorption peak in the low energy region. Furthermore, there was a defined isosbestic point at 500 nm reminiscent of what is commonly observed for an interconverting population of two distinct molecular chromophores.

All of these features suggest that the naphthalene-based conjugated polymers have more localized electronic structures compared to the related annulene-based materials. This can be attributed to the greater energetic cost that must be paid to achieve quinoidal structure that ultimately restricts the extent of intrapolymer resonance delocalization. The naphthalene polymers can be viewed as consisting of simple molecular chromophores that remain isolated along the polymer main chain, therefore presenting molecular characteristics in both the neutral and oxidized states. On the other hand, the annulene polymers do not pay as high of an energetic cost to achieve the quindoidal structure, and we see what can be thought of as a superposition of different absorbing species. This is due to the fact that although the annulene may in principle be capable of supporting a longer effective conjugation length, in practice this may be locally restricted to differing degrees due to conformational biases exerted in the solid state thin films used for spectroelectrochemical analysis. Regardless, this trend does seem to be general (not just limited to thiophene-containing polymers) as evidenced by our recent work with furan-based copolymers, where the annulene and naphthalene copolymers with furan show spectral signatures similar to poly(**MT**) and poly(**NT**), respectively [18]. We are currently preparing and studying soluble variants of these materials to unravel these issues further.

### 2.2. Self-assembling pi-conjugated oligomers for aqueous enviroments

As mentioned above, the intermolecular pi-electron associations among polymeric or oligomeric entities are another important aspect of charge transport through organic electronic materials. Indeed, many elegant model oligomeric structures with defined extents of pi-stacking have been studied in the context of molecular analogies to the pi-dimers and other putative charge carriers involved in energy transport [19,20,21,22,23,24]. Organic semiconductors also rely upon favorable pi-pi interactions in the solid state crystal lattices to achieve conduction in doped states. These studies have also given some design criteria to synthesize electronic molecules with pre-determined degrees of cofacial pi-electron overlap that might otherwise be energetically unfavorable. A notable example is the work of John Anthony who has placed solubilizing silyl acetylene groups onto pentacene and other acenes in order to achieve greater degrees of cofacial pi-stacking [25]. Indeed, this feat of crystal engineering for single crystals of functionalized pentacenes can be extended to large-area domains produced through solution processing or thermal deposition onto electronically relevant surfaces needed to construct transistors or other electronic devices.

We became interested in the construction of one-dimensional supramolecular aggregates of pi-conjugated oligomers as an approach to control the intermolecular orientations of electronic materials at the nanoscale. There are several classes of self-assembling molecules derived from prototypical pi-electron oligomeric materials such as oligophenylenes, oligothiophenes, rylene diimides and others that through proper molecular design are able to self-associate and by doing so lead to the formation of nanoscale one-dimensional (1-D) objects such as cylindrical micelles, ribbons, tubes and bundles [26,27,28,29,30,31]. The general motivation behind these materials is that they allow for the production of nanostructured functional organic electronic materials that could conceivably be manipulated or fashioned into devices much like their inorganic semiconductor nanowire counterparts. However, using lithography to pattern conjugated polymers or oligomers at this length scale (ca. 20 nm and below) is by no means routine, and using chemical synthesis to build the nanostructures would be prohibitively time consuming and limiting in materials quantities.

Despite the tremendous progress in the self-assembly of these 1-D objects in organic solvents, assembly of discrete 1-D organic electronic nanomaterials from aqueous environments has proven quite challenging. Water competes for the hydrogen-bonding synthons commonly used to enforce intermolecular interactions that are not screened to such a dramatic extent in organic solvents. Furthermore, the strongly hydrophobic pi-electron oligomers are not commonly used in aqueous solvents unless charged groups are incorporated on the solubilizing side chains. Nanostructures formed within domains of ionic block copolymers or lyotropic liquid crystals are well-established, but discrete 1-D nanomaterials are not as prevalent as their counterparts formed in organic solutions. A 1-D nanomaterial formed under physiologically relevant conditions would have exciting prospects for nanobiomedicine since these electrically responsive structures would be on the same size scale as the structural components of the extracellular matrix.

Organic fluorophores have found broad use in many protein labeling schemes, where a relevant dye is ligated typically through established amidation chemistry, Michael-type additions, *etc.* Kiick and Galvin used fluorescent dyes to probe through-space pi-electron interactions as mediated by the geometry imposed by the protein α-helices [32]. They used bromo-phenylalanine residues that were site-specifically incorporated as substrates for Heck coupling to ultimately place stilbene chromophores in specific orientations along the helices. MacPhee has used the native Fmoc group attached to the terminus of a small peptide (as present from the solid-phase peptide synthesis) to effect energy migration [33]. Bäuerle and Stupp have recently studied self-assembling peptides that incorporate oligothiophenes as prepared through solution synthesis [34,35]. In our work, we sought to install the pi-conjugated oligomer directly into the peptide backbone using streamlined techniques that would enable all peptide manipulation to be performed on solid-phase supports [36]. The initial approach required the synthesis of pi-conjugated “amino acids” where one end of the pi-system would bear a carboxylic acid and the other end would have an Fmoc-protected amine. The Fmoc amino acid peptide chemistry for solid-phase synthesis is quite established and broadly applied for the construction of oligopeptides.

**Figure 4:**
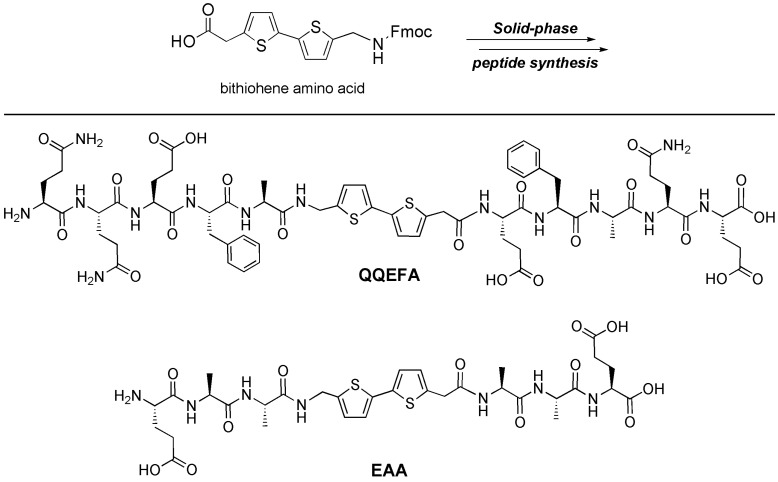
A bithiophene amino acid can be included into peptide backbones through classical Fmoc-based solid-phase peptide synthesis leading to self-assembling peptides such as **QQEFA** and the smaller **EAA**.

As a first test case, we prepared a bithiophene amino acid through a multi-step organic synthesis starting from smaller thiophene building blocks. Importantly, this amino acid could be prepared in gram scale thus providing sufficient quantities needed for peptide synthesis. Using this bithiophene amino acid, we prepared a peptide **QQEFA** that incorporated glutamine-rich peptide flanking sequences as inspired by recent work to prepare β-sheet fibril forming materials (Figure 4) [37]. Although this approach in principle could be used to prepare soluble peptides with backbone embedded units (such as disordered peptides or α-helical peptides), we specifically targeted peptides with strong propensities to self-associate and aggregate. We were also interested in establishing minimal sequences that would also support the assembly scheme. We found that flanking sequences as short as three peptides were sufficient to promote the assembly while still maintaining the aqueous solubility (such as the **EAA** peptide shown). Mono or dipeptides might also be sufficient, and we will probe these materials in the future.

A key element of our initial molecular design was a pH responsive nature that could trigger the assembly. The first generation self-assembling peptides incorporated carboxylic acids that were ionized at basic or even neutral pH. The anionic carboxylates provided sufficient electrostatic repulsion among molecules to effectively prohibit appreciable intermolecular association. When the pH was lowered and the carboxylates protonated, this charge screening led to the establishment of other enthalpic stabilization such as β-sheet hydrogen bonded networks [38]. In practice, solutions of ionizable peptides were fairly fluid while acidification led to the formation of self-supporting hydrogels (or markedly more viscous solutions). This is often empirically associated with cross-linked polymer networks or extended networks derived from the collective interactions of small molecule gelators. In the case of these and other self-assembling peptide constructs, the “cross-links” can be envisioned to arise among the contacts between individual 1-D objects formed upon acidification of the peptide solutions.

**Figure 5:**
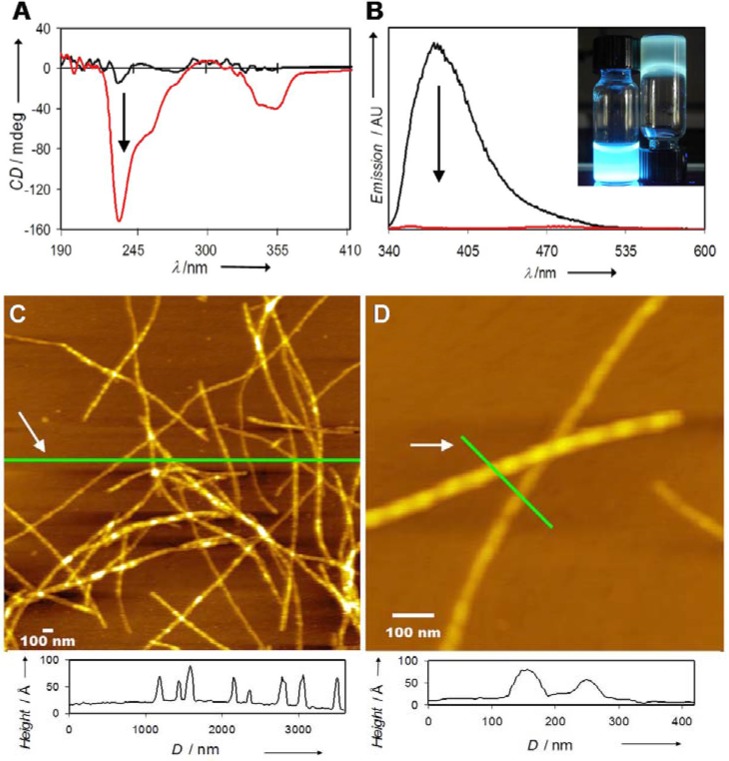
(A) Circular dichroism and (B) fluorescence of **QQEFA** in basic (dissolved, black traces) and acidic (assembled, red traces) aqueous solutions. The inset of (B) depicts a concentrated solution (left) and a gel (right) irradiated at 365 nm. Tapping-mode atomic force microscopy images and height profiles of the indicated line trace of (C) large area and (D) isolated structures formed after assembly and deposition on freshly cleaved mica surfaces. Figure taken from reference [36] with permission (Copyright 2008, American Chemical Society).

Although this empirical evidence is quite compelling, we further verified the assembly process through spectroscopic measurements and through microscopy. One common approach to explore peptide assembly is through circular dichroism (CD). This technique is sensitive to the conformation of a collective population of amide chromophores, with characteristic signatures arising from α-helical, β-sheet or random coil secondary structures. Even more structural subtlety can be extracted, but we feel that caution is warranted when applying these standards to poorly soluble unnatural materials. With our chromophore-embedded materials, the usual spectroscopic signatures were obscured by the chromophore absorption, thus complicating the assignment of a specific β-sheet conformation. As shown in Figure 5A, the CD signal in basic solution was featureless, while acidification and self-assembly were accompanied by a very strong CD response. We interpret this as a sign that the transition moments of the bithiophene π-π* chromophores reside within chiral environments (rather than particular arrangements or chiralities of amide chromophores).

Fluorescence spectroscopy is another tool used to probe the assembly process. When the peptide was molecularly dissolved (here, at basic pH), a strong fluorescence was observed in solution. Acidification of the solution led to a dramatic quenching of the fluorescence. This can be attributed to two factors, the first being specific electronic effects imposed by exciton coupling, whereby certain geometrical orientations of interacting transition dipoles lead to “forbidden” transitions [39,40]. The second is simply an increase in the importance of non-radiative pathways for decay of the photophysically excited state through the conditions imposed in the macromolecular matrix upon the assembly and gelation process. We show in Figure 5B both the spectroscopic and macroscopic manifestations of the fluorescence quenching, and more studies are needed (and are in progress) to understand how the assembly influences the radiative and non-radiative rates of fluorescence decay.

Perhaps the most striking evidence for the self-assembly comes from atomic force microscopy (AFM) of the materials that make up the hydrogels. Samples for analysis were prepared on freshly cleaved mica surfaces by simple deposition of a diluted hydrogel that had been mechanically vortexed to break up the gel and disperse the structural components. AFM revealed the presence of 1-D nanomaterials with a *z*-axis height between 2–6 nm (shown for the assemblies formed form **QQEFA** in Figure 5C, D). It is important to emphasize that the assembly process here does not yield one unique supramolecular architecture, a reflection of the multiple energy sinks possible during the formation of amyloid like materials spanning from initial aggregation and nucleation events to complex fibrils. One could expect ribbons, tapes, and higher-order structures where these tapes stack amongst themselves. However, since the molecular length of the **QQEFA** peptide is approximately 4 nm, we suspect that only early-stage structures are being visualized on the surface. Another unique feature was the presence of superstructural periodicity (shown as about 76 nm, Figure 5D). This feature could be reminiscent of supercoiling phenomena common for many other natural amyloid-based materials as well as for DNA coiling into histones [41]. The origin of this effect in our materials is not clear, but it could arise from an internal strain imposed by the unusual quadrupolar nature of the bithiophene pi-system. With the availability of more complex chromophores that can be incorporated into the self-assembling peptides, we will be able to explore these effects in more depth.

## 3. Conclusions

We have described some of our recent work using conjugated oligomers as electropolymerizable units bearing unusual aromatic rings and as precursors to self-assembled nanostructures in aqueous environments. Both of these approaches have been enabled by the vast wealth of knowledge gained in the preparation of oligomeric species using transition metal-mediated cross-coupling reactions. These oligomers helped to reveal the extents to which unusual Hückel aromatic topologies enhanced the delocalization of charge carriers as deduced with spectroelectrochemistry. The cross couplings also provided amino acids that allowed for the incorporation of hydrophobic pi-conjugated function into aqueous and physiologically relevant environments with the help of ionizable water-soluble oligopeptides. Our future directions with the annulenes are to study their performance as disordered charge transporting materials for semiconductor and photovoltaic applications. With the bioelectronic nanomaterials, we are now working to incorporate bioactive oligopeptide signals covalently onto the self-assembling molecules such that the resulting supramolecular structures both as individual objects or within a suitable hydrogel matrix can serve to elicit a variety of cellular responses.
